# Current Applications of Nanoemulsions in Cancer Therapeutics

**DOI:** 10.3390/nano9060821

**Published:** 2019-05-31

**Authors:** Elena Sánchez-López, Mariana Guerra, João Dias-Ferreira, Ana Lopez-Machado, Miren Ettcheto, Amanda Cano, Marta Espina, Antoni Camins, Maria Luisa Garcia, Eliana B. Souto

**Affiliations:** 1 Department of Pharmacy and Pharmaceutical Technology and Physical Chemistry, Faculty of Pharmacy, University of Barcelona, 08028 Barcelona, Spain; lora_ana@hotmail.com (A.L.-M.); acanofernandez@ub.edu (A.C.); m.espina@ub.edu (M.E.); marisagarcia@ub.edu (M.L.G.); souto.eliana@gmail.com (E.B.S.); 2 Institute of Nanoscience and Nanotechnology (IN2UB), University of Barcelona, 08028 Barcelona, Spain; 3 Centro de Investigación Biomédica en Red de Enfermedades Neurodegenerativas (CIBERNED), University of Barcelona, 08028 Barcelona, Spain; 4 Department of Pharmaceutical Technology, Faculty of Pharmacy, University of Coimbra (FFUC), Polo das Ciências da Saúde, Azinhaga de Santa Comba, 3000-548 Coimbra, Portugal; marianags1994@gmail.com (M.G.); j.dias.ferreira@outlook.pt (J.D.-F.); e_miren60@hotmail.com (M.E.); camins@ub.edu (A.C.); 5 Department of Pharmacology, Toxicology and Therapeutic Chemistry, Faculty of Pharmacy and Food Sciences, University of Barcelona, 08028 Barcelona, Spain; 6 CEB—Centre of Biological Engineering, University of Minho, Campus de Gualtar, 4710-057 Braga, Portugal

**Keywords:** multifunctional nanoemulsions, targeted delivery, cancer

## Abstract

Nanoemulsions are pharmaceutical formulations composed of particles within a nanometer range. They possess the capacity to encapsulate drugs that are poorly water soluble due to their hydrophobic core nature. Additionally, they are also composed of safe gradient excipients, which makes them a stable and safe option to deliver drugs. Cancer therapy has been an issue for several decades. Drugs developed to treat this disease are not always successful or end up failing, mainly due to low solubility, multidrug resistance (MDR), and unspecific toxicity. Nanoemulsions might be the solution to achieve efficient and safe tumor treatment. These formulations not only solve water-solubility problems but also provide specific targeting to cancer cells and might even be designed to overcome MDR. Nanoemulsions can be modified using ligands of different natures to target components present in tumor cells surface or to escape MDR mechanisms. Multifunctional nanoemulsions are being studied by a wide variety of researchers in different research areas mainly for the treatment of different types of cancer. All of these studies demonstrate that nanoemulsions are efficiently taken by the tumoral cells, reduce tumor growth, eliminate toxicity to healthy cells, and decrease migration of cancer cells to other organs.

## 1. Introduction

Nanoemulsions are colloidal dispersions that can be used as drug vehicles mainly used for molecules with low water solubility constituted of safe grade excipients [1,2]. This dosage form is composed of a heterogeneous dispersion of a nanometer droplet in another liquid, which leads to high stability and solubility [3]. The encapsulation protects the drug from degradation and increases its half-life in the plasma [4].

**Figure 1:**
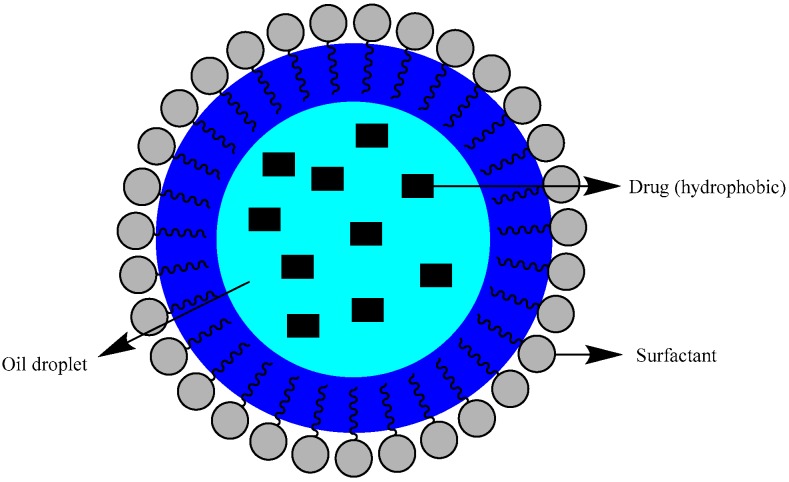
Nanoemulsions structure (based on [5]).

To disperse the droplets in an aqueous phase, emulsifying agents are used to stabilize the system. Emulsifying agents are compounds with an amphiphilic profile that reduce the interfacial tension between two immiscible phases as they are constituted of hydrophobic bicarbonate tails that tend to place themselves in non-polar liquids and a polar head that usually places itself in polar liquids (Figure 1) [5].

The research in cancer therapy has become more focused on nanoemulsions since they hold characteristics essential to achieve an efficient therapeutic effect: large surface area, superficial charge, elevated half-life of circulation, specific targeting, and the imaging capacity of the formulation. Since cancer cells are surrounded by vascularized tissues, nanoemulsions can easily accumulate in these tissues with their size as an advantage for passing through barriers. Above all this, they can also be designed to define their function, encapsulate distinct types of drugs, and select specific targets [6].

The tumor microenvironment is composed of extracellular matrix (ECM), fibroblasts, epithelial cells, immune cells, pericytes, adipocytes, glial cells (present only in the nervous system), proteins, vascular cells, and lymphatic cells [7]. ECM is essential in processes of growth, structure, migration, invasion, and metastasis of the tumor cells which have specific surface markers that can be targeted by drugs. The delivery of oxygen and nutrients to the tumor is achieved by simple diffusion, but when the tumor becomes larger than 2.0 mm^3^, the oxygen levels decrease, leading to hypoxia conditions and angiogenic development of new blood vessels [8]. Therefore, by inhibiting the angiogenic process, cell growth can also decrease. In recent years many anti-angiogenic drugs have been developed: bevacizumab (vascular endothelial growth factor, VEGF-neutralizing antibody), sorafenib (VEGF signaling pathway blockers), sunitinib, and pazopanib. However, these inhibitors of angiogenesis are characterized by marked toxicity, enhanced resistance, and barriers to delivery of compounds [9]. Nanoemulsions can be used to encapsulate the drug inside its core, reducing toxicity and enhancing payload delivery.

Cancer cells get their energy balance using glycolysis. Nonetheless, as a consequence of hypoxia conditions, the final metabolite, pyruvate, is transformed into lactate which is eliminated by a monocarboxilate transporter using H^+^ and generating tumor acidification. The hypoxia environment also increases the expression of carbonic anhydrase IX, resulting in the production of bicarbonate from carbon dioxide (CO_2_) which ends up in the uptake process of the weakly-basic tumor cells, leading to a gradient between the extracellular and intracellular milieu of the tumor. This way, pH-responsive lipids might play an interesting role since they are stable at a pH of 7.4 but can change their chemical behavior when settled in an acidic pH, with further release of the therapeutic load [1].

The disordered and heterogeneous profile of tumors stroma leads to fluctuations in the presence of oxygen, drugs, and essential molecules in the tumor microenvironment [10]. This results in the previously referenced hypoxia and neovascularization leading to metastasis. However, the instability of lymphatic vessels might enhance the retention time of drugs since their clearance rate decreases. This set of effects result in high vascular permeability and low lymphatic drainage, named Enhanced Permeability and Retention (EPR) [11]. Macromolecular and hydrophobic drugs can take advantage of EPR. This therapeutic method is called Passive Targeting [4]. Nanoemulsions with sizes between 20 and 100 nm can be encapsulated and accumulated in tumor tissues, being small enough to pass through blood vessels but big enough to avoid fast renal clearance. Nevertheless, with this range of sizes, the probability of opsonization by the Mononuclear Phagocytic System (MPS) increases [12]. Coating the nanoemulsions with hydrophilic polymers can avoid this problem [13]. Positively charged particles are more likely to be retained through longer periods of time by cancer cells, due to a negatively charged molecule in the tumor cell surface, phosphatidyl-serine [14]. Passive targeting is, however, unable to differentiate healthy tissues from cancerous ones [15]. Active targeting is known as the process through which ligands are associated with the surface of nanoemulsions becoming able to recognize a certain molecule on the tumor tissue. It also takes advantage of the environment surrounding the tumor. What makes it more efficient than passive targeting is the fact that it also generates a new strategy to deliver the drug specifically to cancer cells and, within those, to specific types of cancer cells [16]. The established bond can be of distinct types such as ligand–receptor and antigen–antibody [17]. Active targeting moieties connect to over-expressed receptors in cancer cells like folate [18], transferrin [19], epidermal growth factor (EFGR) [20], or prostate-specific membrane antigen (PSMA) [21]. The targeted delivery causes a specific toxicity in tumor cells and diminished side effects, and is also capable of resorting to surface changes in order to enhance sensitivity to stimuli [22].

The chief mechanisms of multidrug resistance (MDR) are a consequence of the overexpression of multidrug transporters and modifications in the course of apoptosis [23]. Transporter-dependent MDR originates from an overexpression of drug-efflux pumps of the ATP-binding cassette (ABC) family that exports drugs from the cell, removing several anti-cancer drugs. P-glycoprotein (P-gp) encoded by the ABC1 gene was the first ABC transporter identified. It can pump vinblastine, colchicine, etoposide, and paclitaxel (PCX) from the cell [24,25]. Moreover, MDR related with the apoptotic pathway is responsible for enhancing expression of anti-apoptotic genes, such as Bcl-2 and nuclear factor kappa B (NF-kB) [26]. Among other strategies, MDR can be reduced with P-gp inhibitors, with diminishment of Bcl-2 and NF-kB expression, and with nanocarriers (passive or active targeting) [2]. Moreover, more ABC transporters have been described for the resistance of several drugs. In humans, it was estimated that there are 49 ABCs which are ubiquitously distributed in the central nervous system, lung, liver, pancreas, stomach, intestine, and kidney and several anatomical cellular barriers [27].

Small interfering RNA (siRNA) molecules were developed as auxiliary chemotherapy by reducing MDR proteins expression or downregulating anti-apoptotic genes. The problem is that there are few suitable vectors to co-deliver siRNA and drugs. Nanoemulsions might be the solution to this issue, since their combined use with P-gp modulators or Bcl-2 inhibitors can surpass MDR [28]. Ceramides are a family of apoptotic molecules produced in environmental stress situations and they play the role of programmed cell death messenger. There are MDR cells able to avoid apoptosis by over expressing glucosylceramide, responsible for transforming ceramide into its inactive glycosylated form. Ceramide can be delivered by a nanoemulsion with a targeted purpose, increasing apoptotic effects in the tumor tissue [2].

Nanoemulsions can be conjugated with antibodies (Abs) or their fragments for targeting purposes, which is most valuable since antigen–Ab binding is specific and selective. Several studies indicate that this conjugation leads to internalization by cancer cells and successful delivery of drug loaded nanoemulsions. The nanocarrier–Ab complex can be stimuli responsive to make it even more specific to cancer tissues [29]. A process called SELEX provides a library of ssDNA and ssRNA that can be selected to form DNA or RNA oligonucleotides, resulting in aptamers [30]. In comparison to Abs, aptamers have smaller size, no immunogenicity, easy production, and fast penetration. They efficiently bind to the compound of interest and fold into secondary and tertiary DNA/RNA structures. SELEX also allows the selection of aptamers selective for tumor cells based on receptor and biomarker recognition [31]. Folic acid and folate receptors have a large affinity for one another, making folic acid an ideal targeting moiety [32]. Cancers in the brain, lung, pancreas, breast, ovary, cervix, endometrium, prostate, and colon have high folate receptor expression [33], while in normal tissues it is only located on polarized epithelia in the apical surface [34]. Studies indicate that, as cancer progresses, so does the number of folate receptors [35]. Folic acid is not expensive, not toxic, not immunogenic, easy to pair with nanocarriers, and possesses a high binding affinity, being stable both in circulation and storage [36]. Nanoemulsions can also be conjugated with oligonucleotides. However, oligonucleotides are unstable, have a very short half-life in biological fluids, and weak intracellular penetration, making them a rare choice for conjugation with nanoemulsions. Changing phosphodiester to phosphorothionate can raise defenses against enzymatic degradation [37]. Conjugation with polyethylene glycol (PEG) [38], cationic liposomes [39], micelle polyelectrolyte complex (PEC) [40], lipidic and plasmidic DNA complexes [41], and pH-sensitive nanocarriers [42] may help with the remaining problems, enhancing the success of cancer therapy.

The monitoring procedures in cancer treatment with none or very little invasion and tissue damage are possible due to the improvement of imaging techniques. These techniques are often based on the conjugation of nanoemulsions with fluorophores and, seldom, radioisotopes since they can be toxic to humans [2].

## 2. Nanoemulsions—A Brief Overview

### 2.1. Composition of the Nanoemulsions

Nanoemulsions are colloidal dispersions consisting of oil, surfactant, and an aqueous phase. The nanoemulsion core will have an impact on the therapeutic payload of the drug, physico–chemical properties, particle size, and stability [43,44]. The formulation can be formed by long chain triglycerides (LCT) which create larger sized particles, with a diameter of 120 nm, or short chain triglycerides (SCT) leading to smaller particles, around 40 nm. Regarding LCT, soybean oil is very often used due to its high content of essential C18 fatty acids like linoleic acid. Medium chain triglycerides, usually from coconut oil, could be also used alone or in a mixture with LCT to overcome their possible immunosuppressive actions and inhibition of lymphocytes [44,45].

The size of the particles will impact the final aspect of the formulation. In general, the smaller the particle, the higher the stability of the formulation. This particular issue is useful against flocculation, gravitational force, and Brownian motion. However, SCT oils are very soluble in water, facilitating Ostwald’s ripening [46].

The ideal emulsifying agent should reduce interfacial tension, be rapidly adsorbed at the interface, and stabilize the surface by electrostatic or stearic interactions. An emulsifier is an amphiphilic molecule such as surfactants (Tween^®^ 80), amphiphilic proteins (caseinate), phospholipids (soy lecithin), polysaccharides (modified starch), or polymers (PEG) [46]. PEG-modified nanoemulsions are used to enable specific targeting and longer circulation time [18]. Texture modifiers, weighting agents or ripening retarders can also be used [47,48]. Sorbitan fatty acid esters such as Spans^®^ could also be used as non-ionic surfactants [49].

Nanoemulsions responsive to external stimuli can also be developed using temperature and pH-sensible materials aiming the induction of a conformational change in the formulation which furthers the release of the payload [50,51].

### 2.2. Physical and Chemical Characterization of Nanoemulsions

Several physical and chemical properties can influence the behavior of nanoemulsions. Average size is one of the crucial parameters regarding this system [52]. Also, size distribution measured as polydispersity index (PDI), is of extreme relevance since the range of nanoemulsion size should be known to evaluate further biological responses [52]. Both parameters could be measured by dynamic light scattering (DLS) which makes use of the Brownian motion of colloidal particles—since they scatter the light to find the diffusion coefficient of the particle [1].

Moreover, modifications of the cellular response due to surface charge is also a known phenomena [52]. Therefore, surface charge of the nanoemulsions should be characterized. The charge of the system’s surface influences stability and electrostatic interactions and its measurement demands the presence of a magnetic field. The particles’ electrophoretic movement follows Henry’s equation (Equation (1)).

(1) $$\mu \;=\frac{2\varepsilon \zeta f\;\left(ka\right)}{3\eta }$$

where *µ* is the electrophoretic mobility, *ε* the dielectric constant, *ζ* the zeta potential (ZP), *f*(ka) the Henry’s function and *η* the viscosity.

The morphology of the oil droplet is essential in the future definition of the stability of the formulation. The lipophilic–hydrophilic behavior of the nanoemulsion also has significant impact on drug loading, directly influencing the success of drug encapsulation [1]. Optical microscopy, even using differential interference contrast or other phase contrast methods, is generally not a viable method for examining nanoemulsions [33]. However, microscopic techniques are essential in order to obtain reliable data about the actual morphology of the system. In this sense, transmission electron microscopy (TEM) and scanning electron microscopy (SEM) had proven to be useful in order to observe the structure of the nanoemulsions [53]. In addition, SEM gives a three-dimensional image of the droplets [33].

The amount of drug loaded into nanoemulsions constitutes a critical parameter of the formulations aimed to deliver the drug to a target tumoral tissue. In this sense, different approaches in order to carry out the measurement have been performed. It could be measured by separating the free drug from the encapsulated drug and, in this sense, either the free drug could be measured (indirect entrapment efficiency) or the drug encapsulated into the nanoemulsions could be measured by previously dissolving the nanoemulsions core using suitable organic solvents [54]. The separation of the non encapsulated drug from the nanoemulsion could be carried out using a filtration procedure, filtration–centrifugation device, an ultracentrifuge, or dialysis membrane [55,56]. The amount of loaded drug can be measured as the percentage (entrapment efficiency, EE) or as the concentration of drug per nanoemulsion droplet.

### 2.3. Stability Studies

Stability studies are mandatory to characterize the nanoemulsions. An accelerated stability study can be performed by centrifugation of nanoemulsions in such way that the creaming process is accelerated. The traditional approach used to measure the stability of the formulations by is their storage at 4.0 and 25.0 °C for a period of three to six months. Sample characteristics such as average size, polydispersity index, surface charge, and efficiency of encapsulation are usually analyzed once a month. No significant modifications of the nanoemulsion parameters should be measured in order to confirm that the nanometric emulsion is stable under the storing conditions. Moreover, the Food and Drug Administration (FDA) states that stability should be evaluated over long term, intermediate, and accelerated times. The peroxide value (PV), anisidine value (AV) and Total Oxidation Value (TOTOX) are used to assess degradation products and, therefore, the stability of each formulation. The pH value can be modified by oil oxidation processes quantified by the parameters mentioned [1].

### 2.4. Nanoemulsion Drug Release

Drug release process is responsible for drugs bioavailability, absorption, and kinetics. Its evaluation is usually carried out using either Franz diffusion cells or using the dialysis bag method. The latter consists of placing the sample in a dialysis bag and the sample is dialyzed using a buffer in the sink receiver compartment, under stirring conditions at 37 °C [57]. In Franz diffusion cells the nanoemulsion is placed on the donor compartment and it is separated from a receptor chamber (usually filled with phosphate buffer) by a dialysis membrane. The amount of drug that is released from the nanoemulsions to the receptor chamber is analyzed at different time points, obtaining the drug release profile [34].

### 2.5. Nanoemulsion Production

The two phases of nanoemulsion production are the heated and mixed phases (with controlled temperature and agitation) so that the dispersion becomes as homogeneous as possible. Then, the emulsion goes through a process of shear force homogenization to achieve minimal particle size. In the end, particles will have a layer of emulsifiers separating the lipophilic interior from the aqueous phase. This specific layer is a barrier and displays repulsive forces (electrostatic, steric, or electrosteric, depending on the emulsifier) to stabilize the formulation [1].

High shear methods use high pressure homogenizers, microfluidizers, and ultrasonicators [58]. The size of the particle is associated with the instruments and variables like energy, time, temperature, and formulation composition. High energy operations have the plus side of potentially being scaled-up, but they might be inappropriate for certain heat sensitive drugs. In this case, low energy and temperature methods must be used like self-emulsification phase transition and phase inversion [59].

#### 2.5.1. High Pressure Homogenization

**Figure 2:**
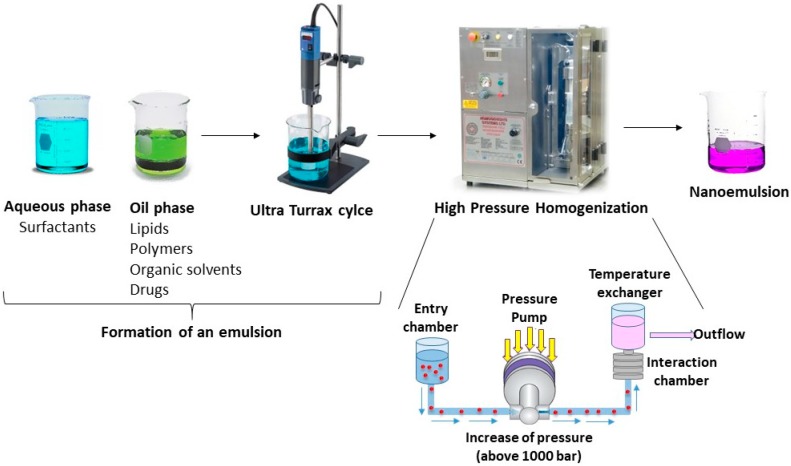
High pressure homogenization technique.

This technique makes use of a high-pressure homogenizer/piston homogenizer to produce nanoemulsions (Figure 2). In the High Pressure Homogenization (HPH) method, an aqueous phase containing the emulsifier is added to an organic phase and ultraturrax is used to form an emulsion. Afterwards, the emulsion is added to the HPH in order to reduce droplet size [60]. Several homogenization cycles and pressures can be applied to obtain the desired nanoemulsion parameters. Therefore, this technique is able to produce small sized particles. Over the course of HPH, a variety of forces contribute to get to a small size of particle: hydraulic, turbulence, and cavitation [1].

The main advantage of this method is that it can be applied several times in order to obtain a suitable droplet size [32,33,59].

#### 2.5.2. Microfluidization

**Figure 3:**
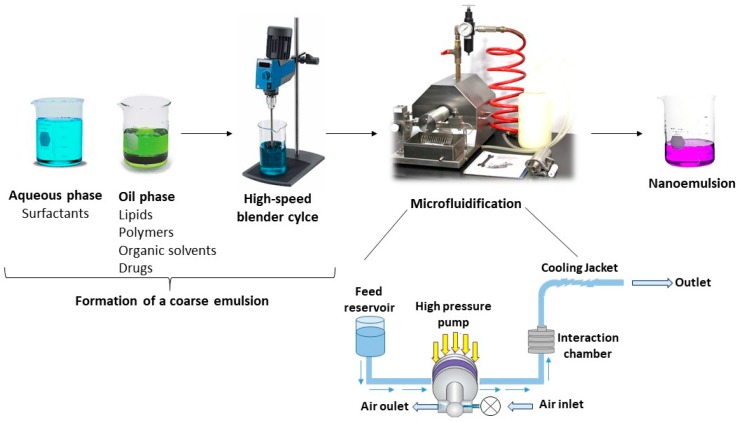
Microfluidification technique.

The microfluidization process requires a microfluidizer instrument [61]. This instrument is patented and consists of a high-pressure positive displacement pump (500–20,000 psi) that makes the product go through the interaction chamber, consisting of small channels (Figure 3). The product flows through the micro-channels on to an impingement area resulting in very fine particles of a submicron range. The two solutions (aqueous phase and oily phase) are combined together [33]. The coarse emulsion is introduced into a microfluidizer where it is further processed to obtain a stable nanoemulsion. The coarse emulsion is passed through the interaction chamber of the microfluidizer repeatedly until the desired particle size is obtained. The bulk emulsion is then filtered through a filter under nitrogen to remove large droplets resulting in a uniform nanoemulsion. This technique can be used in order to produce nanoemulsions at the industrial scale [33].

#### 2.5.3. Phase Inversion Temperature Technique

The phase inversion temperature (PIT) method has highlighted a relationship between minimum droplet size and complete solubilization of the oil in a microemulsion bicontinuous phase independently of whether the initial phase equilibrium is single or multiphase.

Due to their small droplet size, nanoemulsions possess stability against sedimentation or creaming with Ostwald ripening forming the main mechanism of nanoemulsion breakdown [7]. Phase inversion in emulsions can be one of two types: transitional inversion induced by changing factors which affect the hydrophile-lipophile balance (HLB) of the system, e.g., temperature and/or electrolyte concentration, and catastrophic inversion, which can also be induced by changing the HLB number of the surfactant at a constant temperature using surfactant mixtures.

**Figure 4:**
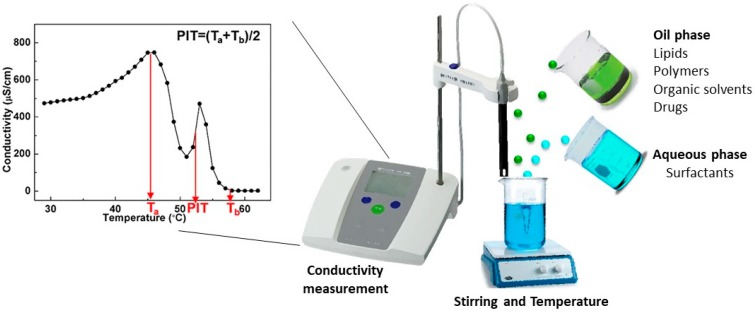
Phase inversion temperature technique (based on [62]).

The PIT method employs temperature-dependent solubility of nonionic surfactants, such as polyethoxylated surfactants, to modify their affinities for water and oil as a function of the temperature (Figure 4) [62]. It has been observed that polyethoxylated surfactants tend to become lipophilic on heating owing to the dehydration of polyoxyethylene groups. This phenomenon forms the basis of nanoemulsion fabrication using the PIT method. In the PIT method, oil, water, and nonionic surfactants are mixed together at room temperature. This mixture typically comprises o/w microemulsions coexisting with excess oil, and the surfactant monolayer exhibits a positive curvature. When this macroemulsion is heated gradually, the polyethoxylated surfactant becomes lipophilic and at higher temperatures, the surfactant gets completely solubilized in the oily phase and the initial o/w emulsion undergoes phase inversion to w/o emulsion. The surfactant monolayer has a negative curvature at this stage. This method involves heating of the components and it may be difficult to incorporate thermolabile drugs, such as tretinoin and peptides, without affecting their stability. It may be possible to reduce the PIT of the dispersion using a mixture of components (surfactants) with suitable characteristics, in order to minimize degradation of thermolabile drugs [33].

#### 2.5.4. Solvent Displacement Method

**Figure 5:**
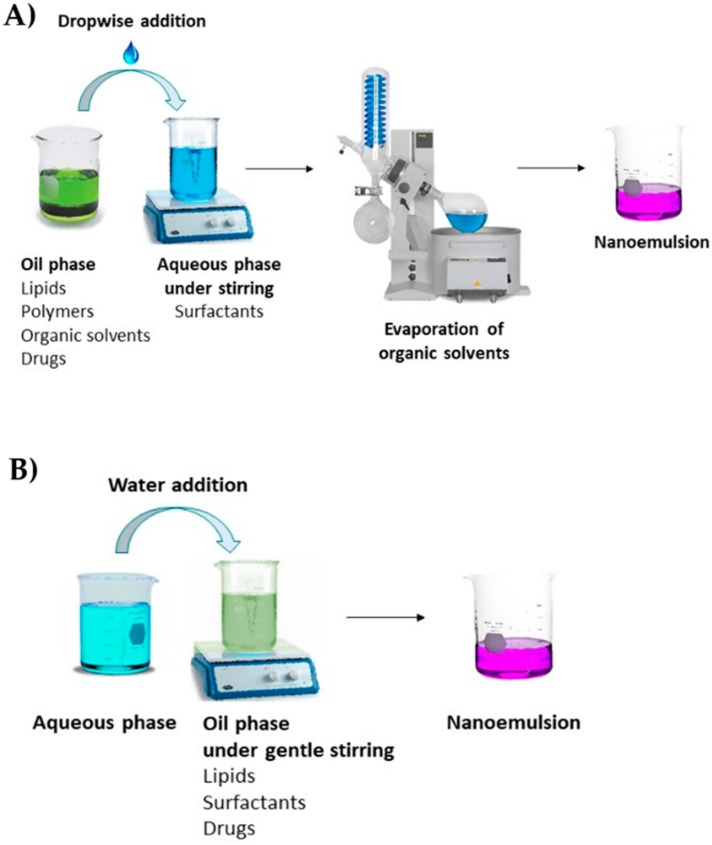
(**A**) The solvent displacement method, and (**B**) the phase inversion composition method.

The solvent displacement method for spontaneous fabrication of nanoemulsions has been adopted from the nanoprecipitation method used for polymeric nanoparticles (Figure 5A).

In this method, the oily phase is dissolved in water-miscible organic solvents, such as acetone, ethanol, and ethyl methyl ketone. The organic phase is poured into an aqueous phase containing surfactant to yield spontaneous nanoemulsions by rapid diffusion of organic solvent [63]. The organic solvent is removed from the nanoemulsions by a suitable means, such as vacuum evaporation.

Solvent displacement methods can yield nanoemulsions at room temperature and require simple stirring for the fabrication. Hence, researchers in pharmaceutical sciences are employing this technique for fabricating nanoemulsions mainly for parenteral use. However, the major drawback of this method is the use of organic solvents, such as acetone, which require additional inputs for their removal from the nanoemulsion. Furthermore, a high ratio of solvent to oil is required to obtain a nanoemulsion with a desirable droplet size. This may be a limiting factor in certain cases. In addition, the process of solvent removal may appear simple at the laboratory scale but can pose several difficulties during scale-up [33]. Therefore, reproducibility and scale-up are the major drawbacks of this method.

#### 2.5.5. Phase Inversion Composition Method (Self-Nanoemulsification Method)

This method generates nanoemulsions at room temperature without the use of organic solvents and without increasing temperature.

Kinetically stable nanoemulsions with small droplet sizes (~50 nm) can be generated by the addition of a water phase into a solution of surfactant in oil, with gentle stirring and at a constant temperature (Figure 5B) [64]. The spontaneous nanoemulsification has been related to the phase transitions during the emulsification process and involves lamellar liquid crystalline phases or D-type bicontinuous microemulsion during the process. Nanoemulsions obtained from the spontaneous nanoemulsification process are not thermodynamically stable, although they might have high kinetic energy and long-term colloidal stability [33].

### 2.6. Metabolism

Upon systemic administration, the nanoemulsions have to escape from the mononuclear phagocitiyc system (MPS) and the renal clearance pathway in order to reach the tumor tissue [65]. The MPS constitutes a biological barrier made by phagocytic cells that could capture nanoemulsions [65].

The nanoemulsion in the bloodstream interacts with erythrocytes, opsonins, monocytes, platelets, leukocytes, dendritic cells, tissue macrophages, Kupffer cells of the liver, lymph nodes, and B cells of the spleen. The formulation is very likely to interact with erythrocytes since they represent the largest fraction of blood cells, resulting in possible hemolysis and removal by macrophages [66]. The extended half-life of this type of formulation increases the chance of interaction with blood cells and, therefore, events of thrombogenicity can occur resulting in blood vessel occlusion [67]. Opsonins can be adsorbed to the surface of nanoemulsions, facilitating the uptake by macrophages, which reduces the drug delivery to the desired place. This activation of immune cells can also result in anaphylactic, allergic, and hypersensitivity reactions [66]. Size, charge, and surface properties of the nanoformulation also influence erythrocytes. Large cationic or anionic particles have a higher tendency to go through phagocytosis. In addition, cationic surfaces are more likely to damage erythrocytes and cause hemolysis [67,68]. Opsonization can be reduced by adding PEG [68], poloxamer [69], or poloxamine [70] to the surface of the nanoemulsion as they create a “steric shield” around the formulation. Cell uptake of nanoparticles is attained through phagocytosis, macropinocytosis, or endocytosis, accumulating in lysosomes, vacuoles, or the cytoplasm [1]. For the formulations aimed to treat brain tumors, the brain–blood barrier (BBB) stands as a great obstacle to drug delivery that can be surpassed with the use of nanoemulsions targeted to reach receptors expressed in the place of action [71].

The main problem in the metabolism of nanoemulsions is the hepatic clearance [72]. Those nanoparticles taken by hepatocytes are eliminated by the biliary system and the ones taken by Kupffer cells are subjected to phagocytosis, degradation, and further elimination. Renal clearance represents an eminent portion of metabolism of nanoemulsions. Glomerural filtration depends on the particle size; particles smaller than 6 nm are filtered in the kidney and the larger ones return to systemic circulation [73].

## 3. Nanoemulsions Applied to Cancer Therapy

### 3.1. Nanoemulsions as a Strategy to Overcome MDR

MDR tumors are a major barrier to effective cancer therapy and, along with metastasis, are estimated to be major contributors to death by cancer. The expression of multifunctional efflux transporters from the ABC gene family have been known to play a crucial role in MDR of tumor cells [25].

ABCs are responsible for the efflux of various endogenous ligands such as proteins, lipids, metabolic products, and drugs such as cytotoxic antibiotics by using energy produced from the hydrolysis of ATP [27].

There are several transporters expressed by the ABC family causing MDR of different antitumoral drugs. P-gp also referred as MDR-1 or ABCB-1, encoded by the ABC1 gene was the first ABC transporter identified and it can pump vinblastine, colchicine, etoposide, and paclitaxel [25,27]. In addition, there are other relevant MDR transporters such as ABCA2 encoded by the *ABCA2* gene, which is for estramustine resistance. MRP1 encoded by *ABCC1* is responsible for doxorubicin, vincristine, etoposide, colchicine, campothethin, and methotrexate resistance and it is one of the most widely expressed transporters in tumoral cells. MRP2 is another transporter of this family and it is responsible for vinblastine, cisplatin, doxorubicin, and methotrexate resistance. It is located on the cell membrane of polarized cells such as kidney, liver, and intestinal epithelium [25,27]. MRP3 encoded by *ABCC3* is responsible for the transportation of organic anions and pumps antitumoral drugs such as methotrexate and etoposide [25,27]. MRP4 encoded by *ABCC4* pumps methotrexate, 6-mercaptopurine, 6-TG 6-thioguanine. In some cases one drug can be the substrate of more than one ABC transporter such as 6-mercaptopurine and 6-TG 6-thioguanine which are also pumped by MRP5 and etoposide which is pumped by MRP6 in addition to MRP1 and MRP3. MRP8 is encoded by *ARCC11* genes and pumps 5-fluorouracil whereas MXR/BCRP (multixenobiotic resistance and breast cancer resistance proteins, respectively) encoded by *ABCG2* genes causes mitoxantrone, topotecan, doxorubicin, daunorubicin, CPT-11, imatinib, and methotrexate resistance [25].

As these data highlight, in oncology, the search for new compounds for the inhibition of these hyperactive ABC pumps is a growing interest in order to increase chemotherapeutic effects. In this sense, several ABC pump inhibitor/modulators functionalizing nanoemulsions have been explored to address the cancer associated MDR [27].

Ganta and colleagues developed nanoemulsions functionalized with folate in order to efficiently deliver docetaxel to ovarian cancer cells overcoming docetaxel MDR [74]. The nanoemulsions were targeted with folate because the folate receptor is poorly expressed the majority of normal tissues. However, it is overexpressed in many cancers especially in epithelial ovarian cancer. In addition, the author’s evaluated P-gp expression and demonstrate that folate receptor mediated endocytosis is capable of bypassing MDR present in ovarian cancer cell lines. Therefore, the nanoemulsions targeted with folate were able to successfully deliver docetaxel by receptor mediated endocytosis that showed enhanced cytotoxicity capable of overcoming ABC transporter mediated taxane resistance [74]. In recent years the taxanes, such as paclitaxel and docetaxel, have emerged as fundamental drugs in the treatment of breast cancer although overcoming MDR is a major issue [75]. In order to overcome this obstacle, Meng and colleagues used baicalein in order to inhibit P-gp and also to increase oxidative stress [76]. Increasing oxidative stress is claimed as a suitable strategy to improve cell sensitivity to paclitaxel due to the fact that cellular reactive oxygen species (ROS) and gluthatione are extremely important for cellular redox reactions. Using this strategy, the author’s co-encapsulate paclitaxel and baicalein in nanoemulsions in order to treat breast cancer. The developed nanoemulsions were able to increase ROS, decrease cellular GSH and enhance caspase-3 activity in MCF-7/Tax cells. More importantly, an in vivo antitumor study demonstrated that baicalein–paclitaxel nanoemulsions exhibited a much higher antitumor efficacy than other paclitaxel formulations. These findings suggest that co-delivery of paclitaxel and baicalein in nanoemulsions might be a potential combined therapeutic strategy for overcoming MDR [76]. A different strategy to overcome MDR of paclitaxel was used by Zheng and colleagues [77]. They aimed to prepare nanoemulsions able to alter the levels of Bax and Bcl-2 expression and also inhibit the P-gp transport function [77]. In order to achieve this goal, they used a vitamin E derivative. Vitamin E is an antioxidant and its mechanisms consist of reducing peroxyl radicals and eliminating the chain reaction of fatty acid radical propagation [77]. It has also been demonstrated that the vitamin E derivative used in this study, TPGS, is one of the most potent and commercially available surfactants that serves as a P-gp inhibitor, and it can reverse MDR in cancer [78]. Vitamin E can disrupt Bcl–xL–Bax interactions, activates Bax, and thus mediates mitochondrial-centered apoptotic cell death. Therefore, vitamin E based nanoemulsions containing paclitaxel were suitable for study of paclitaxel-resistant human ovarian carcinoma cell lines [77].

### 3.2. Nanoemulsions for Different Types of Cancer

#### 3.2.1. Nanoemulsions for Cancer Treatment

Nanotechnology has been shown to be a suitable strategy for cancer treatment and, therefore, researchers focused their efforts on the treatment of several types of cancer. The following section summarizes the most recent advances regarding the most common forms of cancer which can also be observed in Table 1.

#### 3.2.2. Nanoemulsion for Colon Cancer Therapy

Colon cancer represents a large portion of cancer related deaths in the world [79]. Within this category, the subclassification includes familial adenomatous polyposis, hereditary nonpolyposis colorectal cancer, sporadic colon cancer, and colitis-associated cancer [80]. Operation combined with herbs, immunotherapy, radiotherapy, and/or chemotherapy tend to be the choices in colon cancer treatment. Still, survival rate decreases about five years after surgery due to metastasis and recurrence, which means the main cause of death is not the tumor itself [81]. Cancer invasion and migration is possible due to epithelial mesenchymal transition (EMT), a mechanism where epithelial cells transform into mesenchymal cells, changing cell structure and increasing adhesion and migration [82].

Lycopene (LP), present in tomatoes, has several functional features—protection against chronic diseases, anti-proliferation activity against leukemia, and colon cancer cells and further triggering of cell cycle arrest on some tumor cells [83]—and its mechanism could be of use in cancer therapy, if it was not for its low stability and bioavailability [84]. The described study focused on developing a nanoemulsion with LP, in order to find a solution for the presented problem. The nanoemulsion formulation also encapsulates gold nanoparticles (AN). AN act only as a drug carrier but is also available to be incorporated with cell receptor ligands contributed for specific cell targeting [85]. However, AN can become toxic in high doses by promotion of human fibroblast cell migration [86]. This effect can be reduced by incorporating liposomes, polymeric substances, or other lipid-based assemblies, such as LP-derived compounds [87].

In this specific formulation, the oil phase is oil with LP, the water phase is an aqueous AN solution, and the emulsifier is Tween 80^®^. It can be used in a human colon cancer line, HT-29. The evaluation of AN alone, LP alone, and the combination of AN and LP effects on the cell line proves the formulation efficiency [79].

The size of AN impacts toxicity; the lower the size, the stronger the effect on HT-29 cells. The enhancement of the volume of LP incorporated can increase the presence of early apoptotic cells. Both the combined treatment and nanoemulsion increase the levels of early apoptotic, late apoptotic, and necrotic cells. Nevertheless, treatment with nanoemulsions induces apoptotic and necrotic cells. The emulsifier does not impact cells in a significant way, only contributing to nanoemulsion stability. Treatment with nanoemulsions with low AN and LP doses results in low expression of procaspases 3 and 8, and Bcl-2 (tumoral markers), while enhancing Bax and PARP-1 expression, with apoptotic effects on cells [79].

#### 3.2.3. Nanoemulsions for Ovarian Cancer Therapy

Platinum (Pt) chemotherapeutics are used in a vast array of cancer treatments. Carboplatin and cisplatin—molecules containing Pt in their composition—increase survival better than any other ovarian cancer treatment [88]. Pt compounds form intra-strand and inter-strand cross links, shattering DNA structure [89]. The problem with Pt action is related to its effects on cells other than cancer cells, as it also ends up killing healthy cells. Furthermore, cancer cells can develop resistance mechanisms to Pt, like promoting membrane pump presence, or inducing enzymes or DNA repair pathways. Therefore, therapeutics in ovarian cancer always consider whether the tumor is Pt-sensitive or Pt-resistant [90].

Nanomedicinal evolution allowed the design of formulations to overcome toxicity and resistance problems. However, the process is not easy due to Pt properties, particularly due to its lipophilicity [91]. Nanoemulsions can improve delivery and efficiency of Pt-related drugs, since they are able to incorporate enormous amounts of hydrophobic drugs and add specific ligands to their surface in order to achieve a targeted delivery [92]. A nanoemulsion encapsulating myrisplastin (novel platinum Pt-based drug) and C6-ceramide (pro-apoptotic substance) with a surface ligand EGFR-binding peptide and gadolinium (imaging agent) was developed in this study to understand its effect in the following ovarian cancer cells: SKOV3, A2780, and A2780CP [90].

A cytotoxicity screening revealed that SKOV3 cells, expressing epidermal growth factor receptor (EGFR), were resistant to cisplastin presenting an inhibitory concentration, IC_50_, of 18 µM. By encapsulating myrisplatin instead, cytotoxicity increased in a very significant way, both in targeted and non-targeted nanoemulsions. The targeted nanoemulsions possess 2-fold more toxicity in comparison to non-targeted formulations. The biggest change in cytotoxicity occurs when ceramide is also encapsulated, with the combination confirming its synergistic behavior. The targeted nanoemulsion containing ceramide and myrisplatin is 50.5-fold more effective than cisplastin. A2780 and A2780CP (not expressing EGFR) presented more toxic effects with myrisplatin than with cisplatin [90].

Zeng and colleagues developed vitamin E nanoemulsions containing paclitaxel able to modulate the levels of Bac and BCL-2 expression (related to tumor drug resistance) and inhibit the P-gp transport. They assessed their effect in paclitaxel-resistant human ovarian carcinoma cell line A2780. Taxol enhanced the antiproliferation effect and decreased the mitochondrial potential. The authors claim that the association of anticancer drugs with vitamin E derivative multifunctional nanoemulsions could be a suitable solution for cancer multidrug resistance [93].

#### 3.2.4. Nanoemulsion for Prostate Cancer Therapy

The number of deaths related to prostate cancer (PrC) has grown in the last decade with 70% of treated patients facing recurrence and transition to an untreatable state [94]. Cancer stem cells (CSCs) or tumor initiating cells (TICs) are the root of cancer development, metastasis, and resistance to therapies [77]. Studies show that cancer cells expressing CSC markers, specially CD133 and CD4, are not only associated with drug resistance, but also proliferate after therapy [95]. Drug resistance in CSCs might be due to up-regulation of drug efflux transporters, activation of anti-apoptotic pathways, inactivation of apoptotic mechanisms, and more efficient response to DNA damage and repair processes [96].

The problem with prostate cancer therapy is that it targets populations of fast-growing cancer cells but not subpopulations like CSCs. Also, anti-prostate cancer drug development resorts to cell lines with high passage numbers for preclinical studies to evaluate anti-cancer agents. These cell lines end up acquiring genomic and epigenomic properties with low or no match with the original tumor [97]. The research team responsible for this study uses a cell line (PPT2), derived from a prostate cancer patient, with a very low passage number and, by this way, immaturity and stem-like properties are kept. PPT2 cells have genes associated with anti-apoptotic signaling and resistance to drugs, being a perfect model for CSC-targeted therapy studies [98].

One drug that is often used in prostate cancer treatment is Abraxane^®^. Abraxane^®^ is a paclitaxel pro-drug, developed to increase its solubility with human serum albumin-bound nanoparticle formulation. However, paclitaxel has shown problems with MDR cancer cells [96]. A new generation taxoid, SBT-1214, is efficient against drug-resistance. This agent can be conjugated with docosahexaenoic acid (DHA), a natural polyunsaturated fatty acid (PUFA) with high affinity for its main bloodstream transporter (human serum albumin) that helps direct toxicity to cancer. Combination of DHA with paclitaxel resulted in a weak decrease in P-gp and ABC transporters [99]. The DHA–SBT-1214 nanoemulsion formulation developed in this study includes phospholipids and fish oil. The affinity of the drug to fish oil will improve drug encapsulation. It is theorized that the nanoemulsion will act on the CSC-initiated PPT2 cell line, taking advantage of EPR effect and resulting in cancer cell apoptosis [96].

The use of patient-derived CSC enriched PPT2 cells can help in the development of drugs that target cells specific for tumor initiation. Combining DHA with SBT-1214 allows the formulation more time in blood circulation. Encapsulating the conjugated hydrophobic drug in a nanoemulsion formulation results in effective delivery. Thus, surface modification with PEG also enhances the time of drug circulation, which increases accumulation due to the EPR effect. The successful cellular uptake means that the nanoemulsion formulation can deliver its payload more efficiently than the drug solution [96].

#### 3.2.5. Nanoemulsions for Leukemia

Cancer is among the leading causes of death worldwide and leukemia is the most the leading cause of cancer-related death in children. In this context, nanoemulsions have been used as biocompatible systems in order to encapsulate drugs and increase the therapeutic effects by decreasing toxic adverse effects.

Lipid nanoemulsions have been used by several authors as a suitable strategy for drug encapsulation for cancer treatment. Moura and colleagues developed lipid nanoemulsions, able to bind to LDL receptors, with the aim of concentrating the chemotherapeutic agents in tissues with low-density lipoprotein receptor overexpression such as tumoral tissues. The authors encapsulate methothraxete for leukemia treatment and assess them in vitro, as the uptake of the nanoemulsions is significantly higher than the free drug increasing the toxicity against tumoral cells [100].

Winter and colleagues develop nanoemulsions encapsulating chalcones for leukemia and assess them in vitro and in vivo. The authors demonstrate that the developed nanoemulsions cause apoptosis of the cancer cells in vitro showing similar anti-leukemic effects both for the free chalcones and nanoemulsion. However, free chalcones induced higher toxicity in VERO cells than chalcones-loaded nanoemulsions. Similar results were observed in vivo. Free chalcones induced a reduction in weight gain and liver injuries, evidenced by oxidative stress, as well as an inflammatory response [101].

#### 3.2.6. Nanoemulsions for Breast Cancer

Breast cancer accounts for 23% of all newly occurring cancers in women worldwide and represents 13.7% of all cancer deaths. Available chemotherapeutic agents are limited mainly due to the low accumulation of chemotherapeutics at the tumors relative to their accumulation at other organs thus leading to increased toxicities. Several strategies have been developed in order to improve the treatment of this in patients.

Nanoemulsions based on natural compounds could constitute a suitable strategy for breast cancer such as the nanoemulsion developed by Periasamy and colleagues using the essential oil of *Nigella sativa* L [102]. This nanoemulsion shows anti-cancer properties in vitro in MCF-7 breast cancer cells by inducing their apoptosis. This nanoemulsion could be useful for the entrapment of active drugs in order to treat breast cancer [102].

Local administration in addition to C6 ceramide nanoemulsion development has also been used as strategy for breast cancer treatment. The authors target cancers and pre-tumor lesions locally by reducing systemic adverse effects using both nanoemulsion drug delivery and local administration. They developed bioadhesive ceramide loaded nanoemulsions and modified their surface with chitosan. The C6 ceramide concentration necessary to reduce MCF-7 cell viability to 50% (EC50) decreased by 4.5-fold with its nanoencapsulation compared to it in solution; a further decrease (2.6-fold) was observed when tributyrin (a pro-drug of butyric acid) was part of the oil phase of the nanoemulsion. Intraductal administration of the nanoemulsion prolonged drug localization for more than 120 h in the mammary tissue compared to its solution [103]. Natesan and colleagues also used chitosan in order to develop nanoemulsions [104]. They encapsulate camptothecin in nanoemulsions and assess them in vitro and in vivo showing the efficacy of the formulations compared to the free drug [104].

#### 3.2.7. Nanoemulsions for Melanoma

Melanoma is the most serious form of skin cancer causing more than 80% of skin cancer-related deaths. The main problem associated with the treatment of melanoma is low response rate to the existing treatment modalities, which in turn is due to the incomplete response by chemotherapeutic agents and inherent resistance of melanoma cells. Standard treatments for late-stage melanoma and metastatic melanoma usually present poor results, leading to life-threatening side effects and low overall survival. For this reason, newer combinations of anti-melanoma drugs and newer strategies utilizing nanotechnology are being studied, such as the use of nanoemulsions.

Kretxer and colleagues developed lipid nanoemulsions encapsulating paclitaxel that are able to bind to bind to low-density lipoprotein (LDL) receptors, decreasing drug associated toxicity and increasing antitumoral action. Moreover, simvastatin association was also assessed in melanoma bearing mice, demonstrating that this drug associated with paclitaxel nanoemulsions increased antitumoral activity, but not with free paclitaxel. This might due to the fact that statins increase LDL receptor expression and these receptors are responsible for the lipid nanoemulsion internalization [105]. Other authors encapsulated cholesterol derivatives, such as 7-ketocholesterol, into lipid core nanoemulsions and assess them in vivo in a murine melanoma cell line where it was demonstrated that the nanoemulsion decreases the tumor size more than 50%, enlarged the necrotic area, and reduced intratumoral vasculature. The in vitro uptake into tumor cells was LDL-receptor-mediated cell internalization and demonstrated that a single dose of the cholesterol nanoemulsions killed 10% of melanoma cells [106].

A different approach was used by Monge-Fuentes and colleagues, such as the photodynamic therapy using acai oil in nanoemulsion. They used this nanoemulsion as a photosensitizer in vitro and in vivo. NIH/3T3 normal cells and B16F10 melanoma cell lines were treated and presented 85% cell death for melanoma cells, while maintaining high viability in normal cells. Tumor bearing C57BL/6 mice treated with acai oil nanoemulsion showed tumor volume reduction of 82% [107].

#### 3.2.8. Nanoemulsion for Lung Cancer Therapy

Paclitaxel (PTX) is an anticancer drug often used to treat lung, breast, pancreatic, and ovarian cancer. It has the ability to interfere with the breakdown of microtubules during cellular division, leading to apoptosis, mitotic arrest, and inhibition of cell functions [108]. PTX has very low water solubility, which is why many formulations, like Taxol^®^ containing Cremophor-EL^®^ and ethanol, have been developed. However, Cremophor-EL^®^ is known for its toxicity, demanding the investigation of targeting molecules for this formulation [109].

Hyaluronic acid (HA) has been investigated for its use in the active delivery of PTX to cancer cells. It is negatively charged, binding specifically to the cluster of differentiation 44 (CD44), a highly expressed tumor cell marker [110]. The development of a nanoemulsion carrier for PTX and HA—HA-complexed PTX nanoemulsion (HPNs)—has the goal of testing the efficiency of the formulation against tumors expressing CD44 in a non-small lung carcinoma cell line (NCI-H460) [111].

HPN demonstrated great physical–chemical particle properties, with a size that allows a long half-life and zeta potential that is suited to stabilize the formulation, a low polydispersity index confirming homogeneity of the formulation, and the desired spherical morphology. Evaluation of tumor weight showed that both PTX nanoemulsions and HPN reduced tumor growth, however the targeted moiety of HPN, HA, enhances the therapeutic efficiency. Results on body weight reveal no significant changes in groups treated with PTX nanoemulsions and HPN, confirming these therapies are less toxic for healthy tissues [111].

Chang and colleagues study the anticancer activity of the curcuminoid extracts of *Curcuma longa* Linnaeus. They prepared nanoemulsions and explored the inhibition mechanism implicated for the anticancer activity against lung cancer cells. The cell cycle of lung cancer cells was retarded at G2/M for both the curcuminoid extract and nanoemulsion treatments, though the cellular pathway may differ. Among different cancer cell lines, H460 cells show an increased susceptibility to apoptosis compared to A549 cells for both curcuminoid extract and nanoemulsion treatments [112].

### 3.3. Nanoemulsions for Nanotheragnostics

Nanotheragnostics in a new strategy consisting of using the power of nanotechnology for imaging and diagnosis purposes. The aim of this strategy is to produce nanoscale agents affording both therapeutic and diagnostic functions [113].

This strategy is associated with different nanotechnological approaches such as nanoemulsions and researchers have focused with special emphasis on cancer treatment/diagnosis [113]. The recent advancements in this field have enabled the characterization of individual tumors, prediction of nanoemulsions–tumor interactions, and the creation of nanomedicines for individualized treatment [113].

In this sense, Fernandes and colleagues developed perfluorohexane nanoemulsions as novel drug-delivery vehicles and contrast agents for ultrasound and photoacoustic imaging of cancer in vivo, offering higher spatial resolution and deeper penetration of tissue when compared to conventional optical techniques. This NE provides a non-invasive cancer imaging and therapy alternative for patients [56].

Wu and colleagues used a similar strategy, developing magnetic nanoemulsions hydrogels inducing magnetic tumor regression based on a ferrofluid-based magnetic hyperthermia of cancers [114]. Moreover, Niravkumar et al. developed nanoemulsions encapsulating three difatty acid platins, dimyrisplatin, dipalmiplatin, and distearyplatin. They developed fatty acid nanoemulsions that selectively bind the folate receptor α (FR-α) and utilize receptor mediated endocytosis to deliver Pt past cell surface resistance mechanisms (FR-α is overexpressed in a number of oncological conditions including ovarian cancer) [115]. Roberts and colleagues used sonophore molecules for multi-spectral optoacoustic tomography (MSOT) of tumors. They combined near-infrared and highly absorbing dyes loaded into nanoemulsions, enabling the non-invasive in vivo MSOT detection of tumors.

### 3.4. Clinical Trials

Nanoemulsions could constitute a suitable alternative to ensure a better treatment for cancer patients. However, as can be observed in Table 2, just a few clinical trials have been reported using these colloidal carriers [121].

Nanoemulsions have been used for superficial basal cancer cell photodynamic therapy in an on-going clinical trial [122]. It compares three photosensitizers using randomized prospective double blinded design (phase 2). The photodynamic therapy is combined with methylaminolevulinate (MAL/Metvix^®^) or with hexylaminolevulinate (HAL/Hexvix^®^) and aminolevulinic acid nanoemulsion (BF-200 ALA/Ameluz^®^) in superficially growing basal cell carcinomas. BF-200 ALA nanoemulsion contains 7.8% of 5-aminolevulinic acid, the first compound in the porphyrin synthesis pathway. It has been formulated with soy phosphatidylcholine and propylene glycol to increase its affinity with epidermal tissue [123]. This formulation has been employed in several clinical trials for the treatment of lentigo maligna (ClinicalTrials.gov ID: NCT02685592), multiple actinic keratosis (ClinicalTrials.gov ID: NCT01893203) and actinic keratosis (ClinicalTrials.gov ID: NCT01966120 and NCT02799069).

As reported earlier, curcumin has been extensively studied for cancer. Curcumin is a natural polyphenolic compound extracted from the rhizomes of *Curcuma longa* and shows different biological activities in an antioxidant and anti-inflammatory capacity, among others [124]. To date, no clinical trial aimed directly to cancer therapy has been undertaken. Instead, curcumin loaded nanoemulsions are being assessed in a randomized, double blinded phase 1 controlled study (ClinicalTrial.gov ID: NCT03865992) [125]. Curcumin nanoemulsions aim to reduce the joint pain in breast cancer survivors with aromatase inhibitor-induced joint disease. Curcumin nanoemulsions will be administered orally to women who have primary invasive adenocarcinoma of the breast taking a third-generation aromatase inhibitor. This anticancer drug has a wide incidence of skeletal adverse events such as bone loss and arthralgia [126].

A second clinical trial using curcumin nanoemulsions has been described for the treatment of women with obesity and high risk for breast cancer (ClinicalTrial.gov ID: NCT01975363). This constitutes a randomized pilot study aimed to determine the tolerability, adherence, and safety of different doses (50 or 100 mg) of nanoemulsion curcumin in obese women at high risk for developing breast cancer. Due to the anti-inflammatory activity of curcumin in breast tissue and fat, the risk of developing breast cancer may be reduced [127].

## 4. Nanoemulsions in the Drug Delivery Field

Nanotechnology has notably improved safety and effectiveness of cancer therapy by developing drug delivery systems such as nanocarriers. Due to their nanometric size, they are suitable for chemotherapeutic passive targeting via the enhanced permeability retention (EPR) effects. Moreover, it can achieve active targeting by receptor-mediated uptake to specific cell types and host tissues [128]. Besides this, it provides a controlled release, an increase of drug stability, and solves water solubility problems related to hydrophobic drugs [129]. Among these nanostructured systems, polymeric nanoparticles, nanostructured lipid carriers, liposomes, and nanoemulsions have shown to be a great approach to achieve drug delivery for cancer treatment [128]. Despite the decades of research about the medical use of inorganic nanoparticles, they have demonstrated a lack of safety and biocompatibility [130]. There are some recently investigations in which overcome this drawback by modifying the particle surface with biocompatible molecules; nevertheless, it needs more research for developing more effective coatings and drug delivery strategies [131]. In contrast, the main advantages of nanoemulsion are its composition. It is formulated using biocompatible components and generally recognized as safe (GRAS) and its easy to scale-up and manufacture [132]. In addition to the advantages mentioned above, common with most nanosystems, nanoemulsions possess a high encapsulation capacity for hydrophobic drugs, great physicochemical stability, potentially improved bioavailability, and the drug pharmacokinetics show lower inter- and intra-individual variability [104,133,134,135]. This system is available for many administration routes. By oral administration, nanoemulsions could protect drug molecules from gastric and gut wall degradation and avoid first-pass metabolism. Nanoemulsions possess a stability similar to liposomes, ethosomes, or microspheres but they possess the advantage of enhanced solubility and absorption of poorly bioavailable molecules [136]. An in vivo study which compared the accumulation in the brain of lipid nanoparticles and nanoemulsions showed that nanoemulsions enhanced the retention time in a significant manner compared to lipid nanoparticles [137]. Moreover, comparative studies about the effect on skin permeation between liposomes, solid lipid nanoparticles, and nanoemulsions have demonstrated that solid lipid nanoparticles tend to release the drug in superficial skin layers, while liposomes and nanoemulsions are able to permeate to deeper skin layers. However, the encapsulation into nanostructure lipid carriers offers increased protection for photosensitive drug than nanoemulsions [138]. In the same way, a nebulized lipid-based nanoemulsion for lung cancer treatment was carried out to explore the possibility of dissolving a large amount of hydrophobic drugs and to increase the resistance towards hydrolysis and enzymatic degradation [139].

## 5. Limitations of Nanoemulsions

Nanoemulsions can be of great utility in the delivery of drugs to cancer cells due to the fact that these systems have been demonstrated to be safe and able to deliver the drug to the target tissue, increasing drug effects and avoiding toxicity. To the present, no formulation of this type has been approved by the FDA. A variety of concepts are decisive since they can limit the success of a system [2].

The production of this formulation might involve high temperature and pressure conditions, depending on the drug and excipients. Therefore, not all types of starting materials are suitable for some particular manufacturing processes. When this happens, the design of an appropriate production method, or even optimization of an already existing one, might be necessary and take a great deal of time. It is crucial to guarantee that labile drugs are viable and can be produced at a larger scale. Conceiving multifunctional nanoemulsions in large scale production might be particularly hard as there is a fair number of variables to consider [2]. To investigate a suitable method for a particular nanoformulation, the material safety, scale-up, and all parameters of quality control need to be taken into account [1].

The system of nanoemulsion and its moieties’ behavior and even its in vivo metabolism need to be carefully evaluated [2]. Every substance behaves in a unique way and so does the metabolism. Absorption, distribution, and excretion can affect parameters like efficiency and safety of drugs and need to be in continuous evaluation [2]. In this sense, targeting nanoemulsions is a major issue for cancer drug delivery since it has been reported that only 0.7% of the drug dose using nanotechnological based strategies is found to be delivered to the solid tumor [65]. In this sense, metabolism is a crucial factor since only the nanoemulsions able to escape from the MPS and renal clearance biological barriers have the opportunity to interact with the tumor tissue [65].

Every time a new material is considered in a formulation, its long-term stability and safety have to be studied. The problem is related to the fact that models used to evaluate toxicity are frequently questionable since results might not be valid due to the absence of real dynamic interactions, normally acting in real human tissues, in a real human body. Research often begins with cellular models or animal species with characteristics and metabolisms that differ from the human body in very important aspects. Also, the way in which an organism behaves varies from person to person and within the person’s state, depending on sex, race, age, environmental features, and a lot of other conditions. All of this complicates the translation from in vitro/in vivo to real-life treatment [1].

Due to these fact, the main limitation of nanoemulsions for cancer drug delivery relies on the low clinical translation of the formulations [65].

## 6. Future Perspectives

Nanoemulsions are drug delivery systems able to encapsulate hydrophilic and hydrophobic molecules designed in order to satisfy a variety of requests [2].

The main challenge of nanoemulsions in future developments is to keep finding mechanisms to improve nanoemulsion efficiency, differentiating them from other formulations. This continuous process of research has to keep in mind the interactions of the drug with the other components in the system, the impacts of the manufacturing mechanism, and drug stability. Along with production, the interaction of nanoemulsions with target cells is also a main study point in future drug development, exploring different ways to induce drug release and uptake. Different routes of administration for nanoemulsions carrying cancer drugs can also be investigated. The key thinking is to come up with new insights on nanoformulation, creating new opportunities for anticancer drug delivery.

The use of nanoemulsions as imaging agents is emerging, as it provides real time monitoring of cancer with minimum destruction and invasion. Traditional imaging techniques involve X-ray tomography, magnetic resonance, and ultrasound and they are all based on marking a targeting nanoemulsion with a radioactive isotope or a fluorophore [1].

Also, in late development are vaccine carriers in nanoemulsion formulations to target tumors. Nanoemulsions, as previously described here, are able to deliver macromolecules, such as antigens which can lead to a useful antigen specific response from the immune system. Nanoemulsions allow a long circulation time and uptake by cells with the specific antibody on their surface for that antigen, or vice-versa, resulting in a highly specific interaction [2].

## 7. Conclusions

Nanoemulsions represent a new and promising strategy in cancer therapy. The employment of a hydrophobic core allows the encapsulation of lipophilic drugs, coming up with a solution for one of the main problems related to cancer treatment drugs. The presence of an emulsifying agent and GRAS excipients allow the engineering of a stable and safe alternative. They are composed by small sized particles, allowing them to be retained for a long time in circulation.

The main advantage of nanoemulsions, in relation to other drug carriers, is that they can be designed to target tumor cells and avoid MDR. This is a significant development in cancer therapy, since its major problem remains in the fact that most anti-cancer drugs fail due to their marked toxicity in healthy cells/tissues or even because cancer cells end up developing mechanisms to resist treatment.

Passive targeting delivery takes advantage of the ERP effect, typical in tumor tissues. However, active targeting might bring even more positive features to the formulation, as it uses not only the EPR effect but also specific targeting moieties for cancer cells. Multifunctional nanoemulsions can co-encapsulate, or bind to their surface, compounds that fight MDR mechanisms.

The examples described in this paper demonstrate diverse methodologies through which nanoemulsions can be designed to achieve successful therapeutic outcomes in several types of cancer.

All of these positive features are useless if the manufacturing process and the metabolism of the drug and excipients are not carefully evaluated. These two parameters represent the chief barriers in nanoemulsion development. To achieve this type of formulation, go through every phase of clinical trials, and be approved, all variables must be considered and innovative solutions have to be studied in order to create anticancer drugs that are safe and efficient. The development of this type of formulation is critical in cancer therapy as this multifactorial illness results in a sizable portion of deaths and no completely viable therapy has been found to this day.

## Figures and Tables

**Table 1:** Summary of some recent nanoemulsions developed and applications for cancer.

Nanoemulsion Constituents	Active Compound	Production Technique and Physicochemical Parameters	Type of Cancer	Therapeutic Efficacy and Other Observations	Ref.
Nanoemulsions carrying gold nanoparticles Tween 80^®^	Lychopene	Production using ultrasonication method; Average size: 25.0 nm; Zeta potential: −32.2 mV	Colon cancer	Nanoemulsions reduced the expressions of procaspases 8, 3, and 9 and PARP-1 and Bcl-2; Nanoemulsions enhanced Bax expression; Nanoemulsions increase HT-29 cell apoptosis and reduced their migration capability; Upregulation of epithelial marker E-cadherin and downregulation of Akt, nuclear factor kappa B, pro-matrix metalloproteinase (MMP)-2, and active MMP-9 expressions.	[79]
EGFR-targeted nanoemulsion; EGFR binding peptide; Lipidated gadolinium (Gd) chelate; Lipidated EGFRbp; Egg lecithin; PEG2000DSPE; Glycerol	Myrisplatin: novel platinum pro-drug; C6-ceramide: pro-apoptotic agent	High shear microfluidization process; Average size: <150 nm; Stable in plasma for 24 h	Ovarian cancer	Efficacy was 50-fold drop in the IC_50_ in SKOV3 cells as compared to cisplatin alone; Improved efficacy over cisplatin nanoemulsions.	[90,116]
Vitamin E nanoemulsions: composed of α-TOS, and vitamin E Brij 78 and TPGS	Paclitaxel	Preparation using emulsification–evaporation method; Average size: 236.7 nm; Polydispersity index: 0.29; Zeta potential: −23.9 mV; Drug loading: ≈1.04%	Multidrug resistance cancers	30% of paclitaxel is release in vitro for the first 24 h; Nanoemulsions increase Bax cell levels of and decrease Bcl-2 expression and inhibit the transport function of P-gp and decrease mitochondrial potential in paclitaxel-resistant human ovarian carcinoma cell line A2780/Taxol.	[93]
Taxoid pro-drug nanoemulsions: Lipoid E80^®^; Polysorbate 80; DSPE-PEG2000	DHA-SBT-1214 (omega-3 fatty acid conjugated taxoid pro-drug)	Production using HPH technique; Average size: 228 ± 7 nm; Zeta potential: −27 mV; Entrapment efficiency: 97%	Prostate cancer	Nanoemulsion surface was modified with PEG; Weekly intravenous administration of nanoemulsions in mice bearing subcutaneous PPT2 tumor xenografts suppressed tumor growth compared to Abraxane^®^; Nanoemulsions show significant activity against prostate CD133high/CD44+/high tumor-initiating cells in vitro and in vivo.	[96]
Lipid nanoemulsion: mixture of phosphatidylcholine, triolein, and cholesteryloleate	Didodecyl methotrexate (ddMTX, esterification reaction between methotrexate and dodecyl bromide)	Production using ultrasonication method; Average size: 60 nm; Entrapment efficiency: 98%; Nanoemulsions were stable at 4 °C for 45 days	Leukemia	After 48 h of incubation with plasma, approximately 28% ddMTX was released; Nanoemulsion uptake by neoplastic cells was higher than free methotrexate which resulted in markedly greater cytotoxicity; Nanoemulsions cytotoxicity against neoplastic cells was higher than free methotrexate.	[100]
Lipid nanoemulsions: Miglyol 812; Lipoid S75; Polysorbate 80	Chalcone	Production using ultrasonication method; Average size: 110 nm; PI: 0.17; ZP: −19 mV, 93% EE	Leukemia	Nanoemulsions maintained the antileukemic effect of chalcones; Nanoemulsions decreased chalcone toxic effects in non-tumoral cells and in animals.	[101]
Hyaluronic acid complexed nanoemulsions: DL-α-tocopheryl acetate; Soybean oil; Polysorbate 80; Ferric chloride	Paclitaxel	Production using HPH technique; Average size: 85.2 nm; ZP: −35.7 mV; EE: ≈100%	Lung cancer	Hyaluronic acid nanoemulsions inhibited tumor growth, probably because of the specific tumor-targeting affinity of HA for CD44-overexpressed cancer cells.	[111]
Lipid nanoemulsion (7KCLDE): Egg phosphatidylcholine; Triolein; Cholesteryl oleate; Cholesterol	7-ketocholesterol	Average size: 20–50 nm	Melanoma	Single 7KCLDE injection killed ≈10% of melanoma cells; 7KCLDE was injected into B16 melanoma tumor-bearing mice, was accumulated in the liver and tumor. In melanoma tumor in mice 7KCLDE promoted a >50% tumoral size reduction, enlarged the necrotic area, and reduced intratumoral vasculature. 7KCLDE increased the survival rates of animals, without hematologic or liver toxicity.	[106]
Folic acid targeted albumin nanoemulsions; Albumin; Folic acid; Poloxamer 407	Carbon monoxide releasing molecule-2 (CORM-2)	Production using HPH technique; Average size: <100 nm	Lymphoma	Nanoemulsions increased survival of BALB/c mice bearing subcutaneous A20 lymphoma tumors.	[117]
Perfluorohexane nanoemulsions	Perfluorocarbon (contrast agent)	Production using ultrasonication method; Average size: <100 nm; Suitable long-term stability	Ultrasound and photoacoustic imaging of cancer in vivo	Higher spatial resolution and deeper tissue (compared to conventional optical techniques); Non-invasive cancer imaging and therapy alternative for patients.	[115]
Carotenoid; Nanoemulsions; CapryolTM 90; Transcutol^®^HP; Tween 80	Carotenoid extract from *Lycium barbarum* L.	Production using ultrasonication method; Average size: 15.1 nm	Colon cancer	Nanoemulsions release carotenoids in the acidic environment (characteristic of tumors) but not at physiological pH; Nanoemulsions IC50 of 4.5 μg/mL; Nanoemulsions upregulate p53 and p21 expression and down-regulate CDK2, CDK1, cyclin A, and cyclin B expression and arrest the cell cycle at G2/M in HT-29 colon cancer cells.	[118]
Perfluorocarbon nanoemulsions; Perfluorodecalin; Fluorinated poly(ethylenimine)	siRNA to silence the expression of Bcl2 gene	Production using ultrasonication method (for nanoemulsions); Formation of polyplexes using nanoemulsions and siRNA; Average size: ≈150 nm; ZP: +50 mV; One week stability	Melanoma	Nanoemulsions-based polyplexes induced apoptosis and inhibited tumor growth in a melanoma mouse model; Nanoemulsions-based polyplexes showed potential for in vivo ultrasound imaging.	[119]
Curcumin nanoemulsions; Medium chain tryglicerides; Cremophor RH40; Glycerol	Curcumin	Self-microemulsifying method; Average size: 34.5 nm; Polidispersity index: 0.129; ZP: −8.54 mV	Prostate cancer	Curcumin nanoemulsions enhance the cellular cytotoxicity, cellular uptake, cell cycle arrest, and apoptosis against prostate cancer cells.	[120]

**Table 2:** Clinical trials using nanoemulsions for cancer drug delivery.

ClinicalTrial.gov ID	Active Compound	Nanoemulsion Constituents	Sponsor and Collaborators	Description	Status	Ref.
NCT02367547	5-Aminolevulinic acid	Soy phosphatidyl-choline; Propylene glycol	Joint Authority for Päijät-Häme Social and Health Care; Tampere University; University of Jyvaskyla	Photodynamic therapy against superficial basal cell cancer.	Active, not recruiting	[122]
NCT03865992	Curcumin	Data not available	City of Hope Medical Center; National Cancer Institute (NCI)	Oral curcumin nanoemulsion for joint pain reduction in breast cancer survivors caused by treatment with aromatase inhibitors.	Recruiting	[125]
NCT01975363	Curcumin	Data not available	Ohio State University Comprehensive Cancer Center	Oral curcumin nanoemulsion to modulate pro-inflammatory biomarkers in plasma and breast adipose tissue.	Active, not recruiting	[127]
